# Risk assessment for type 2 diabetes mellitus and its association with knowledge and health beliefs among university students in three Arab countries

**DOI:** 10.1038/s41598-026-41511-5

**Published:** 2026-03-27

**Authors:** Hebatalla Abdelmaksoud Abdelmonsef Ahmed, Ahmed Yousef, Shady Mohamed Abdelwahab, Mohamed Adel Youssef, Samah Shalaby Elnozahy, Reema Saeed Almater, Lamia Ahmed Saddah, Methqal Al-Dhawi, Noor Ebrahim Naji Al-matari, Mohammed Al-azab, Hoda Ali Ahmed Shiba

**Affiliations:** 1 Public Health & Community Medicine Department, Faculty of Medicine, Kafr-Elsheikh University, Kafr El Sheikh, Egypt; 2 Public Health & Community Medicine Department, Damietta Faculty of Medicine, Al-Azhar University, Cairo, Egypt; 3 Faculty of Medicine, Kafr-Elsheikh University, Kafr El Sheikh, Egypt; 4 Faculty of Medicine, King Khalid University, El-Qassem, Kingdom of Saudi Arabia; 5 Faculty of Medicine, Sana’a University, Sana’a, Yemen; 6 Faculty of Medicine, Al-azhar University, Cairo, Egypt; 7 Public Health & Community Medicine Department, Faculty of Medicine, Al-Azhar University, Cairo, Egypt

**Keywords:** Type 2 diabetes, University students, Health beliefs, Diabetes knowledge AUSDRISK, Arab countries, Diseases, Endocrinology, Health care, Medical research, Risk factors

## Abstract

Type 2 diabetes mellitus (T2DM) is a growing concern in the Arab world, with university students increasingly at risk due to lifestyle factors. This study aims to assess T2DM risk, knowledge, and beliefs among university students aged ≥ 18 years in Egypt, Saudi Arabia, and Yemen. A multinational, cross-sectional study was conducted from January to April 2025 among university students aged ≥ 18 years in Egypt (lower-middle-income), Saudi Arabia (high-income), and Yemen (low-income). A multistage, stratified purposive sampling technique was used to recruit students equally from medical and non-medical faculties in at least three universities per country. Data were collected via structured, interview-based questionnaires consisting of four components: (1) socio-demographic and anthropometric data; (2) the Diabetes Knowledge Questionnaire-18 (DKQ-18); (3) the Health Belief Model Scale (HBMS); and (4) the validated Arabic version of the Australian type 2 diabetes risk assessment tool (AUSDRISK). Among 2787 university students, the mean age was 21.0 years, with the majority being female (74.9%), and enrolled in medical faculties (58.2%). Diabetes knowledge was significantly higher in Egyptians (72.6% with good knowledge) compared to Saudis (63.9%) and Yemenis (59.8%) (*p* < 0.001). Health beliefs followed a similar trend, while diabetes risk was significantly higher among Yemeni students (22.0% with severe risk; *p* < 0.001). Multiple regression analysis revealed that a higher academic year was a positive predictor of both diabetes knowledge (β = 0.155, *p* < 0.001) and health beliefs (β = 0.152, *p* < 0.001), and was negatively associated with diabetes risk (β = − 0.061, *p* < 0.001). Medical faculty enrollment was also positively associated with knowledge (β = − 0.411 for non-medical faculty, *p* < 0.001) and beliefs (β = − 0.228 for non-medical faculties, *p* < 0.001). Parental education and income were positively associated with knowledge and beliefs. Specifically, father’s higher education predicted greater knowledge (β = 0.047, *p* < 0.001), and household income was a significant predictor of both knowledge (β = 0.042, *p* < 0.001) and beliefs (*p* < 0.001). In contrast, diabetes risk was independently associated with male gender (β = 0.305, *p* < 0.001), older age (β = 0.123, *p* < 0.001), higher BMI (β = 0.284, *p* < 0.001), and Yemeni nationality (β = 0.080, *p* < 0.001). Marked differences in diabetes risk, knowledge, and beliefs were observed among students from the three countries. Interventions targeting high-risk groups are essential to improve diabetes awareness and prevention in young adults. Countries like Yemen may benefit from urgent preventive measures, while countries with moderate risk profiles may focus on health promotion and sustained education to avert future disease burdens.

**Supplementary Information:**

The online version contains supplementary material available at 10.1038/s41598-026-41511-5.

## Introduction

Type 2 diabetes mellitus (T2DM) is a chronic metabolic disorder that has reached epidemic proportions globally, posing a major public health challenge due to its chronic complications and increasing economic burden^1^. According to the International Diabetes Federation (IDF), approximately 10.5% of adults aged 20–79 years were living with diabetes in 2021, with this number projected to rise to 46% by 2045^2^. The Arab world, in particular, is witnessing a sharp rise in T2DM prevalence, with approximately 43.2 million adults in the Arab region, making it one of the highest-incidence areas globally, attributed to urbanization, lifestyle changes, unhealthy dietary habits, and low levels of physical activity^3^. In Saudi Arabia, the situation is alarming, with a rapidly increasing prevalence and a trend toward younger age. Studies report a pooled prevalence of T2DM of around 25.0% in adults, with projections indicating continued growth^4^. Moreover, the IDF report for 2024 estimated T2DM prevalence at 22.4% of the adult Egyptian population^5^. In Yemen, it’s estimated to be around 9.6%^6^.

T2DM accounts for more than 90% of all diabetes cases and remains a major contributor to global morbidity and mortality, ranking as the ninth leading cause of death worldwide^7^. Its economic impact is equally substantial. The 2021 IDF report estimated global diabetes-related health expenditures at USD 966 billion, representing 9% of all adult healthcare spending, with middle-income countries carrying a proportionally greater financial burden than high-income nations^8^. In Saudi Arabia, approximately 13.9% of the national health expenditure was allocated to diabetes management, underscoring the significant economic burden posed by the disease^9,10^.

Early identification of T2DM in early adulthood is increasingly important as epidemiological trends reveal a rising incidence of T2DM among individuals under 20 years of age^11^. University students, who are adapting to newfound independence and academic pressures, represent a particularly vulnerable group^12^. Their lifestyle patterns often include unhealthy dietary habits, physical inactivity, smoking, alcohol use, and heightened stress—all of which increase susceptibility to metabolic disorders, especially when coupled with non-modifiable factors such as a family history of diabetes^13^.

Evidence from Arab countries further highlights this vulnerability, with high rates of overweight, obesity, and insufficient physical activity reported among university students, with some studies reporting up to half of students being overweight or obese and more than 50% not meeting recommended physical activity levels^14–17^. Beyond these behavioral and environmental risks, early-onset T2DM is biologically more aggressive; young individuals experience a rapid decline in β-cell function—approximately 25–30% annually, compared with about 7% in adults—leading to accelerated disease progression and the earlier development of complications^18^. These concerns are intensified by the fact that 20–50% of people with T2DM remain undiagnosed globally, particularly in low- and middle-income countries^19^.

Therefore, detecting T2DM early or identifying at-risk young adults before clinical onset, along with fostering strong diabetes knowledge and positive health beliefs^20^, is crucial. Prioritizing these preventive efforts in university-aged populations thus offers a critical opportunity to reduce long-term morbidity and improve health outcomes^21^. This study aimed to assess the risk of developing T2DM among apparently healthy university students aged ≥ 18 years in three Arab countries representing different economic contexts: Egypt (lower-middle-income), Saudi Arabia (high-income), and Yemen (low-income) by employing a standardized and validated AUSDRISK tool with evaluations of the level of diabetes-related knowledge and health beliefs.

## Methods

### Study design and setting

This multinational cross-sectional study was conducted among young adults university students (aged ≥ 18 years) from three Arab countries: Egypt, Saudi Arabia, and Yemen, from January 2025 to April 2025. These countries were purposefully selected to represent varying income levels according to the World Bank’s 2024–2025 income classification, with Egypt representing lower-middle-income, Saudi Arabia representing high-income, and Yemen representing low-income contexts^22^.

### Study population and sampling

The study population included university students aged ≥ 18 years. To enhance the representativeness of the sample, aiming to capture a diverse student population with varying academic, social, and geographic backgrounds, a multistage, stratified purposive sampling technique was employed. In each participating country, research assistants were strategically distributed across a minimum of three different universities. Within each university, faculties were categorized into two main groups based on their academic discipline: medical and non-medical faculties. Research assistants were instructed to recruit participants equally from both categories, ensuring that approximately 50% of the collected sample in each university came from medical faculties and the remaining 50% from non-medical faculties. Excluded participants were those been previously diagnosed with Type 1 or Type 2 diabetes mellitus or pregnant women with gestational diabetes. Trained research assistants approached participants through institutional networks, student groups, and faculty contacts, and conducted data collection using structured, interview-based questionnaires.

### Sample size

The sample size was calculated using G*Power software version 3.1 (Franz Faul, Germany), assuming a 50% prevalence of T2DM risk (as no prior national estimate was available across the three studied countries), a 95% confidence level, and a 5% margin of error. This yielded a minimum required sample of 385 participants per country. After adjusting for a design effect of 1.5 and a 10% non-response rate, the final target sample was approximately 645 participants per country, resulting in a total sample size of 1935 across the three countries. In total, 2787 university students were recruited.

### Data collection tool

Data were collected through a face-to-face interview guide questionnaire with respondents to determine the risk of diabetes among apparently healthy university students. Students were given a clear and concise explanation of the study’s objectives. *The questionnaire consists of four parts*.

*The first part* includes socio-demographic and personal data, including; sex, age (years), weight (kg), height (cm), country, type of residence (urban/rural), type of faculty (medical or non-medical), academic grade, income, father’s occupation and education level, and mother’s occupation and education level.

*The second part* is the validated Diabetes Knowledge Questionnaire-18 (DKQ-18), which assesses participants’ knowledge of diabetes, consisting of 18 items covering five domains: diabetes etiology and symptoms, intermediate nursing, complications, diet and treatment, and elementary nursing. Each item had three possible responses: Yes, No, and I do not know. Items were scored as correct (1 point) or incorrect (0 points). The total score ranged from 0 to 18, with scores ≥ 9 indicating good knowledge and scores < 9 reflecting poor knowledge. The DKQ-18 has been validated for both reliability and accuracy, making it the recommended tool for assessing diabetes knowledge^23^. In the present study, α = 0.835.

*The third part* is the Health Belief Model Scale (HBMS) developed by Schwab et al. to evaluate the participants’ beliefs and attitudes toward diabetes prevention and management^24^. It demonstrated good reliability and validity in a previous Turkish validation study^25^. This scale includes 33 items across five subscales: perceived susceptibility (4 items), perceived seriousness (3 items), perceived benefits of preventive behaviors (7 items), perceived barriers to adopting health behaviors (11 items), and recommended health behaviors (10 items). The scale is a Likert scale, and the items are scored on a three-point scale (1 corresponding to completely disagree and 3 corresponding to totally agree with reverse coding of negative belief statements). Higher scores indicate positive health beliefs, and lower scores indicate negative health beliefs. Each subscale mean was calculated by dividing the total score of its items by the number of items within that subscale. Similarly, the overall mean score of the entire scale was obtained by dividing the combined total score of all items by the total number of items. In the present study, α = 0.891.

*The fourth part* is the validated Arabic version of the Australian Type 2 Diabetes Risk Assessment Tool (AUSDRISK tool), which assesses nine risk factors: age, sex, family history of diabetes, history of high blood glucose, hypertension, smoking, fruit and vegetable intake, physical activity, and waist circumference (Table S1). The total score of the AUSDRISK Arabic version was scaled into the following categories (mild risk: ≤ 4 points, moderate risk: 5–10 points, and severe risk: ≥ 11 points). A cut-off point of 13 was selected to determine the risk of developing DM in the coming five years with sensitivity and specificity of 86.11% and 73.35%, respectively, with a 0.887 (95% confidence interval (CI): 0.824–0.95) area under the curve (AUC), p-value < 0.001^26^.

*Anthropometric measurements*: Waist circumference was measured by wrapping a measuring tape gently around the respondent’s waist at the level of the top of the hip bone. Specific cutoff points of 102 cm for men and 88 cm for women were used to identify risk^27^. The tape was positioned parallel to the ground and formed a complete circle around the trunk. Body mass index (BMI) was calculated using the formula: BMI = weight (kg) / height (m²). This involved dividing the body weight in kilograms by the square of the height in meters. BMI categories were defined as follows: a BMI below 18.5 classified as underweight, a BMI from 18.5 to less than 25 considered within the healthy weight range, a BMI between 25.0 and less than 30 indicating overweight, and a BMI of 30.0 or higher denoting obesity^28^.

All anthropometric measurements were conducted by trained research assistants in each of the three participating countries. Prior to data collection, the researchers in each country held standardized training sessions to ensure uniformity in measurement techniques. These sessions included demonstrations on locating anatomical landmarks, correct positioning of the measuring tape, ensuring the tape was parallel to the floor, and avoiding excessive tension during measurement. The same model of non-elastic measuring tape was used across all sites. Additionally, weight and height were measured following standard procedures. Participants were assessed while wearing light clothing and no shoes. Weight was measured using a calibrated digital scale placed on a firm, level surface, with participants standing still and distributing their weight evenly on both feet. Height was measured using a stadiometer. Participants stood upright with heels together, legs straight, arms at their sides, and head positioned in the Frankfort horizontal plane. The stadiometer headpiece was gently lowered to make firm contact with the crown of the head. All measurements were taken twice, and the average value was recorded to minimize measurement error.

### A pilot test and tools validation

Although the DKQ-18 and the HBMS are internationally validated tools, their application in a different cultural context with a different language necessitated revalidation. An expert panel of four public health professors and two endocrinology professors reviewed the relevance and language clarity of the items, ensuring both face and content validity. For pilot testing, we conducted it on 30 participants from each country to assess the study tools for clarity, feasibility, applicability, and the time required to complete the questionnaires. According to the pilot study results, necessary modifications and improvements were made prior to data collection. The data collection time was approximately 15–20 min. The response rate was 96%.

### Data management and analysis plan

Data were analyzed using IBM SPSS Statistics version 22.0. Descriptive statistics were used to summarize the study variables. Categorical variables were presented as frequencies and percentages, while continuous variables were described using means and standard deviations or medians and interquartile ranges (IQR), if data were not normally distributed (verified using the Shapiro–Wilk test). To compare continuous and ordinal outcomes across the groups, the Mann-Whitney U-test and Kruskal–Wallis test were applied. Effect sizes were calculated and interpreted as follows: epsilon squared (ε²) for Kruskal–Wallis tests (the interpretation values are: 0.01- < 0.06 (small effect), 0.06 - < 0.14 (moderate effect), and > = 0.14 (large effect))^29^ and rank-biserial correlation (r) for Mann–Whitney tests (the interpretation values are: values around 0.10 as small, 0.30 as moderate, and 0.50 or above as large)^30^. For comparisons of categorical variables, the Chi-square test of independence was used. The strength of significant associations was assessed using Cramér’s V (the interpretation values are: ≤ 0.2 (small effect), 0.2 - ≤ 0.6 (moderate effect), and > 0.6 (large effect))^31^.

In evaluating determinants of dependent variables, multiple linear regression analyses were performed. Before running the regression models, the assumptions of linearity, independence of errors, homoscedasticity, and absence of multicollinearity were checked and met. Regression results were reported as standardized beta coefficients (β), and p-values. A p-value < 0.05 was considered statistically significant. Importantly, to account for differences in actual sample sizes across sex, as AUSDRISK scoring inherently includes sex-based risk points (e.g., males are assigned higher baseline scores due to their elevated risk for T2DM, AUSDRISK scores were considered weighted by sex in both descriptive summaries and comparative analyses. The weights were calculated by dividing the target sample size per sex group by the actual number of participants from each sex group. The items of the Validated Arabic Version of AUSDRISK were illustrated in Table S1, Supplementary file.

## Results

Table 1 shows the socio-demographic and personal characteristics of the study participants (*N* = 2787). The mean age of the students was 21.0 years, with the majority being female (74.9%) and residing in urban areas (72.3%). More than half of the participants were enrolled in medical faculties (58.2%). In terms of geographic distribution, 41.1% were from Egypt, 35.4% from Saudi Arabia, and 23.5% from Yemen. Regarding BMI status, 54.7% were in the normal range, while 23.4% were overweight and 10.0% were obese. Fathers’ educational levels were mostly university or postgraduate (42.9% and 21.0%, respectively), while mothers’ educational attainment was lower, with 12.6% and 28.4% having only primary or secondary education, respectively. Most mothers were housewives (61.7%), and nearly half of the fathers (47.1%) worked in governmental jobs. Household income varied, with 12.1% reporting insufficient income and 38.1% reporting sufficient income with the ability to save.

**Table 1:** Socio-demographic and personal characteristics of the studied university students (N=2787).

Studied variables		(N = 2787)	%
Age (Years): *Mean ± SD*		21.0 ± 2.0	
Gender	Female	2088	74.9%
	Male	699	25.1%
Weight (Kg): *Mean ± SD*		63.2 ± 16.3	
Height (cm): *Mean ± SD*		162.7 ± 10.2	
BMI (kg/m2)	Total average *Mean ± SD*	23.8 ± 5.7	
	Underweight (< 18.5 kg/m^2^)	341	12.2%
	Normal weight (18.5- <25 kg/m^2^)	1525	54.7%
	Overweight (25- <30 kg/m^2^)	641	23.0%
	Obese (> 30 kg/m^2^)	280	10.0%
Country	Egypt	1145	41.1%
	Saudi Arabia	986	35.4%
	Yemen	656	23.5%
Residency	Rural	773	27.7%
	Urban	2014	72.3%
Faculty	Medical	1622	58.2%
	Non-medical	1165	41.8%
Academic year	1st year	502	18.0%
	2nd year	452	16.2%
	3rd year	647	23.2%
	4th year	415	14.9%
	5th year	585	21.0%
	6th year	186	6.7%
Father’s occupation	Government worker	1314	47.1%
	Private worker	755	27.1%
	Unemployed	718	25.8%
Father’s education	Illiterate	100	3.6%
	Primary education	220	7.9%
	Secondary education	687	24.7%
	University education	1195	42.9%
	Postgraduate education	585	21.0%
Mother’s occupation	Government worker	900	32.3%
	Private worker	167	6.0%
	Housewife	1720	61.7%
Mother’s education	Illiterate	255	9.1%
	Primary education	350	12.6%
	Secondary education	792	28.4%
	University education	1063	38.1%
	Postgraduate education	327	11.7%
Income	Enough with savings	1063	38.1%
	Enough without savings	1388	49.8%
	Not enough	336	12.1%

SD: Standard deviation; BMI: Body mass index.

Table 2 presents a comparison between students from the three countries regarding their diabetes knowledge (DKQ-18), health beliefs (HBMS), and diabetes risk (AUSDRISK). Diabetes knowledge was significantly higher among Egyptian students, with a median score of 11.0, compared to 10.0 among Saudi and Yemeni students (*p* < 0.001; ε² = 0.031). The percentage of students with good knowledge was highest in Egypt (72.6%), followed by Saudi Arabia (63.9%) and Yemen (59.8%). Health Belief Model scores also differed significantly between countries. Egyptian students had the highest total HBMS scores, followed by Saudis and then Yemenis (*p* < 0.001; ε² = 0.031). Regarding diabetes risk, AUSDRISK scores were significantly higher among Yemeni students (median (IQR): 7.0 (5.0–10.0)) compared to Egyptians (median (IQR): 6.0(3.0–9.0)) and Saudis (median (IQR): 6.0(3.0–8.0)) (*p* < 0.001). Furthermore, a larger proportion of Yemeni students (22.0%) were classified as having a severe risk compared to Egyptians (15.3%) and Saudis (14.5%) (*p* < 0.001, V = 0.114). Details on country-based differences in AUSDRISK components and scores among study participants were shown on Table S2.

**Table 2:** Comparison of diabetes knowledge (DKQ-18), health belief model scores (HBMS), and AUSDRISK scores among the studied university students (N=2787).

Studied variables		Total	Egypt	Saudi Arabia	Yemen	P-value	Effect size
		N = 2787	N = 1153	N = 963	N = 671		
DKQ-18 score	Total, *Median (IQR)*	10.0(7.0–13.0)	11.0(8.0–14.0)	10.0(6.0–12.0)	10.0(7.0–12.0)	< 0.001^*b^	ε^2^=0.031
	Good knowledge, *n (%)*	1808(64.9%)	837(72.6%)	579(60.1%)	392(58.4%)	< 0.001^*a^	V = 0.137
	Poor knowledge, *n (%)*	979(35.1%)	316(27.4%)	384(39.9%)	279(41.6%)		
HBMS score	Total, *Median (IQR)*	2.7(2.5–2.8)	2.8(2.7–2.9)	2.8(2.5–2.9)	2.7(2.5–2.8)	< 0.001^*b^	ε^2^=0.031
	Perceived susceptibility, *Median (IQR)*	2.5(2.2–2.7)	2.5(2.3–2.8)	2.5(2.0-2.8)	2.5(2.0-2.8)	< 0.001^*b^	ε^2^=0.017
	Perceived seriousness, *Median (IQR)*	2.3(2.0–3.0)	2.3(2.3-3.0)	2.3(2.0–3.0)	2.3(2.0–3.0)	0.242^b^	ε^2^=0.001
	Perceived benefits, *Median (IQR)*	3.0(2.7-3.0)	3.0(2.7-3.0)	3.0(2.7-3.0)	2.9(2.6-3.0)	< 0.001^*b^	ε^2^=0.015
	Perceived barriers, *Median (IQR)*	2.7(2.3–2.9)	2.8(2.6–2.9)	2.8(2.3–2.9)	2.6(2.3–2.8)	< 0.001^*b^	ε^2^=0.031
	Recommended health behaviors, *Median (IQR)*	3.0(2.8-3.0)	3.0(2.8-3.0)	3.0(2.8-3.0)	2.9(2.7-3.0)	< 0.001^*b^	ε^2^=0.036
AUSDRISK score	Total, *Median (IQR)*	6.0(4.0–9.0)	6.0(3.0–9.0)	6.0(3.0–8.0)	7.0(5.0–10.0)	< 0.001^*b^	ε^2^=0.031
	Mild risk, *n (%)*	942(33.8%)	407(35.0%)	326(43.2%)	209(24.1%)	< 0.001^*a^	V = 0.114
	Moderate risk, *n (%)*	1366(49.0%)	578(49.7%)	320(42.3%)	468(53.9%)		
	Severe risk, *n (%)*	478(17.2%)	178(15.3%)	109(14.5%)	191(22.0%)		

*Significant, IQR: Interquartile range, a: Chi-squared test, b: Kruskal-Wallis test, V: Cramér’s V effect size, ε^2^: Epsilon-squared, DKQ-18: Diabetes Knowledge Questionnaire-18, HBMS: Health Belief Model Scale, AUSDRISK: Australian Type 2 Diabetes Risk Assessment Tool.

N.B. AUSDRISK scores were weighted by sex to ensure balanced representation when comparing across countries.

**Fig. 1:**
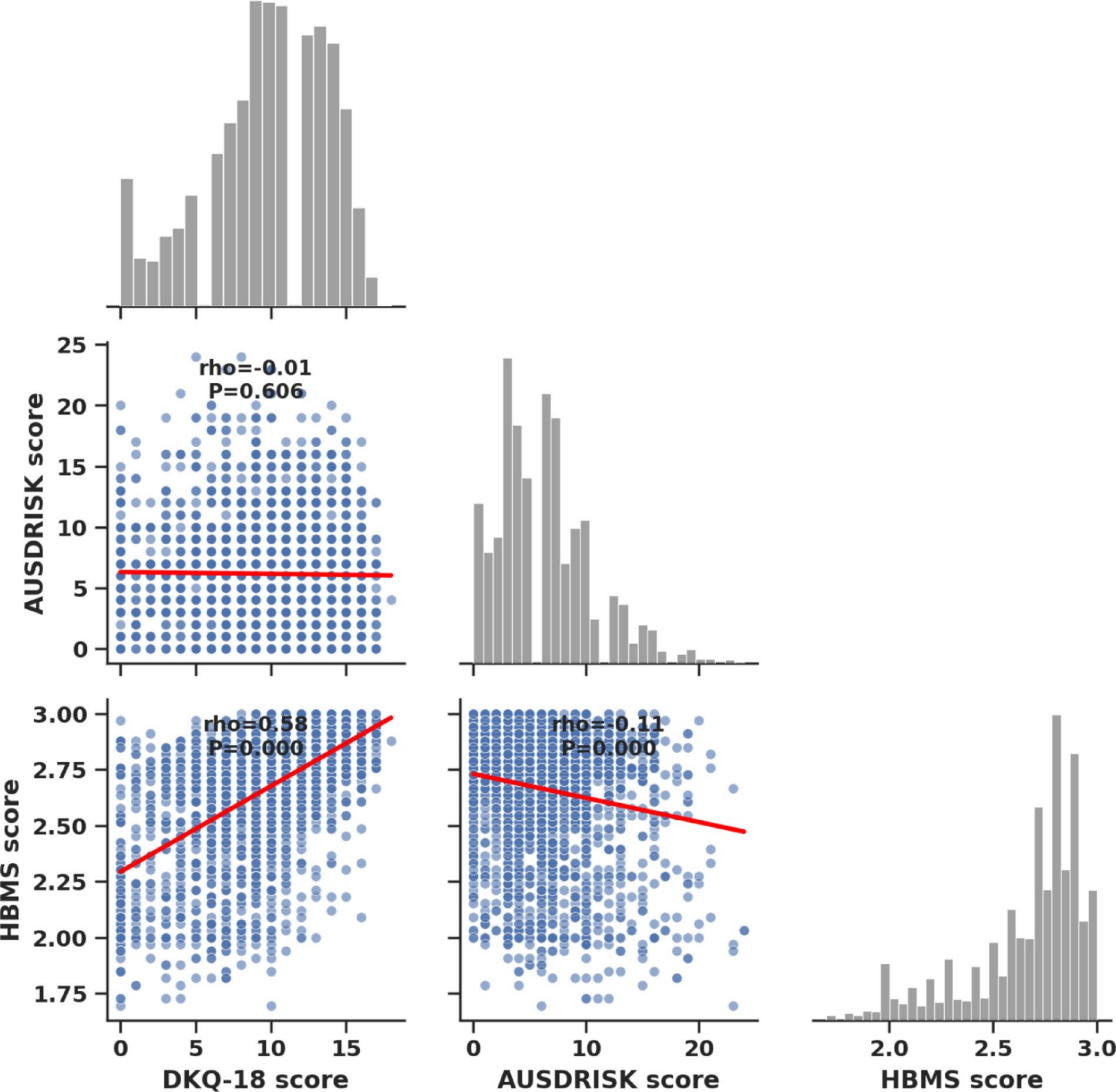
Scatter matrix of AUSDRISK scores in relation to diabetes knowledge (DKQ-18) and health belief model scores (HBMS). DKQ-18: Diabetes Knowledge Questionnaire-18, HBMS: Health Belief Model Scale, AUSDRISK: Australian Type 2 Diabetes Risk Assessment Tool.

Figure 1 shows the Spearman’s rho correlation coefficients between DKQ-18 total score, AUSDRISK total score, and total HBMS score. There was a moderate positive correlation between diabetes knowledge and health beliefs (rho = 0.577, *p* < 0.001). A weak and statistically non-significant negative correlation was found between diabetes knowledge and AUSDRISK scores (rho = − 0.010, *p* = 0.606). Moreover, a small but statistically significant negative correlation was observed between total health belief scores and AUSDRISK scores (rho = − 0.111, *p* < 0.001).

Table 3 describes the distribution of diabetes knowledge scores across sociodemographic subgroups. Students in higher academic years had significantly better knowledge (*p* < 0.001; ε² = 0.050), as did those studying in medical faculties (median (IQR): 12.0(9.0–14.0)) compared to non-medical students (median (IQR): 8.0(5.0–10.0)) (*p* < 0.001; *r* = 0.474). Rural residents scored better than urban residents (*p* = 0.012; *r* = 0.048). Higher parental education, particularly fathers with postgraduate education, was associated with higher knowledge scores (*p* < 0.001; ***ε²*** = 0.023). Students from households with sufficient income and the ability to save had significantly higher knowledge than those with insufficient income (*p* < 0.001; ε² = 0.012).

**Table 3:** Comparison of diabetes knowledge (DKQ-18 Scores) across sociodemographic subgroups among the studied university students (N = 2787).

Studied variables		DKQ-18 score (N = 2787)		
		Median (IQR)	Good knowledge, *n (%)*	Poor knowledge, *n (%)*
Gender	Female	10.0(7.0–13.0)	1364(65.3%)	724(34.7%)
	Male	10.0(7.0–13.0)	444(63.5%)	255(36.5%)
	P value, effect size	0.078^b^, r = 0.033	0.387^a^, V = 0.016	
BMI (kg/m2)	Underweight (< 18.5 kg/m^2^)	10.0(7.0–12.0)	217(63.6%)	124(36.4%)
	Normal weight (18.5- <25 kg/m^2^)	10.0(7.0–13.0)	984(64.5%)	541(35.5%)
	Overweight (25- <30 kg/m^2^)	10.0(7.0–13.0)	419(65.4%)	222(34.6%)
	Obese (> 30 kg/m^2^)	10.0(8.0–13.0)	188(67.1%)	92(32.9%)
	P value, effect size	0.354^c^, ε^2^=0.001	0.798^a^, V = 0.019	
Residency	Rural	10.0(8.0–13.0)	531(68.7%)	242(31.3%)
	Urban	10.0(7.0–13.0)	1277(63.4%)	737(36.6%)
	P value, effect size	0.012^b^*, r = 0.048	0.009^a^*, V = 0.050	
Faculty	Medical	12.0(9.0–14.0)	1311(80.8%)	311(19.2%)
	Non-medical	8.0(5.0–10.0)	497(42.7%)	668(57.3%)
	P value, effect size	< 0.001^b^*,r = 0.474	< 0.001^a^*, V = 0.394	
Academic year	1st year	9.0(6.0–11.0)	291(58.0%)	211(42.0%)
	2nd year	9.0(6.0–12.0)	250(55.3%)	202(44.7%)
	3rd year	11.0(8.0–13.0)	460(71.1%)	187(28.9%)
	4th year	11.0(8.0–13.0)	276(66.5%)	139(33.5%)
	5th year	10.5(7.0–13.0)	386(66.0%)	199(34.0%)
	6th year	12.0(9.0–14.0)	145(78.0%)	41(22.0%)
	P value, effect size	< 0.001^c^*, ε^2^=0.050	< 0.001^a^*, V = 0.140	
Father’s occupation	Government worker	11.0(8.0–13.0)	896(68.2%)	418(31.8%)
	Private worker	11.0(8.0–13.0)	502(66.5%)	253(33.5%)
	Unemployed	9.0(6.0–12.0)	410(57.1%)	308(42.9%)
	P value, effect size	< 0.001^c^*, ε^2^=0.018	< 0.001^a^*, V = 0.097	
Father’s education	Illiterate	9.0(6.0–10.0)	49(49.0%)	51(51.0%)
	Primary education	9.5(6.0–12.0)	130(59.1%)	90(40.9%)
	Secondary education	10.0(7.0–13.0)	418(60.8%)	269(39.2%)
	University education	10.0(7.0–13.0)	789(66.0%)	406(34.0%)
	Postgraduate education	11.0(8.0–13.0)	422(72.1%)	163(27.9%)
	P value, effect size	< 0.001^c^*, ε^2^=0.023	< 0.001^a^*, V = 0.110	
Mother’s occupation	Government worker	11.0(8.0–13.0)	645(71.7%)	255(28.3%)
	Private worker	10.0(7.0–12.0)	1059(61.6%)	63(37.7%)
	Housewife	10.0(7.0–13.0)	104(62.3%)	661(38.4%)
	P value, effect size	< 0.001^c^*, ε^2^=0.011	< 0.001^a^*, V = 0.098	
Mother’s education	Illiterate	10.0(7.0–12.0)	153(60.0%)	102(40.0%)
	Primary education	9.0(6.0–12.0)	201(57.4%)	149(42.6%)
	Secondary education	10.0(7.0–13.0)	494(62.4%)	298(37.6%)
	University education	11.0(7.5–13.0)	725(68.2%)	338(31.8%)
	Postgraduate education	11.0(8.0-13.5)	235(71.9%)	92(28.1%)
	P value, effect size	< 0.001^c^*, ε^2^=0.012	< 0.001^a^*, V = 0.096	
Income	Enough with savings	11.0(8.0–13.0)	725(68.2%)	338(31.8%)
	Enough without savings	10.0(7.0–13.0)	889(64.0%)	499(36.0%)
	Not enough	9.0(6.0–12.0)	194(57.7%)	142(42.3%)
	P value, effect size	< 0.001^c^*, ε^2^=0.012	0.001^a^*, 0.069	

*Significant, ^a^: Chi-squared test, ^b^: Mann-Whitney test, ^c^: Kruskal-Wallis test, r: Rank-biserial correlation, V: Cramér’s V effect size, ε^2^: Epsilon-squared, DKQ-18: Diabetes Knowledge Questionnaire-18.

Table 4 illustrates the variation in HBMS scores across different participant characteristics. Female students reported significantly higher total health belief scores (median = 2.8) than males (median = 2.7) (*p* < 0.001; *r* = 0.133). Students from medical faculties also demonstrated stronger health beliefs (*p* < 0.001; *r* = 0.383), with consistent superiority across all subscales, including perceived susceptibility, seriousness, benefits, and barriers. HBMS scores increased with academic year (*p* < 0.001; ε² = 0.039). Rural residents had higher scores compared to their urban counterparts (median = 2.8 vs. 2.7). Higher educational attainment and employment of parents were positively statistically associated with HBMS scores. Household income also showed a significant gradient, with those from higher-income households demonstrating stronger beliefs (*p* < 0.001; ε² = 0.012).

**Table 4:** Comparison of health belief model scale (HBMS) across sociodemographic subgroups among the studied university students (N = 2787).

Studied variables		HBMS score (*N* = 2787)					
		Total score	Perceived susceptibility	Perceived seriousness	Perceived benefits	Perceived barriers	Recommended health behaviors
		*Median (IQR)*	*Median (IQR)*	*Median (IQR)*	*Median (IQR)*	*Median (IQR)*	*Median (IQR)*
Gender	Female	2.8(2.6–2.8)	2.5(2.3–2.8)	2.3(2.0–3.0)	3(2.7-3.0)	2.8(2.4–2.9)	3.0(2.8-3.0)
	Male	2.7(2.5–2.8)	2.5(2.0-2.8)	2.7(2.3-3.0)	2.9(2.6-3.0)	2.7(2.3–2.9)	2.9(2.7-3.0)
	P value, effect size	< 0.001^b^*, r = 0.133	0.005^b^*, r = 0.071	0.125^b^, r = 0.038	< 0.001^b^*, r = 0.125	< 0.001^b^*, r = 0.114	< 0.001^b^*, r = 0.197
BMI (kg/m2)	Underweight (< 18.5 kg/m^2^)	2.7(2.5–2.8)	2.5(2-2.8)	2.3(2.0–3.0)	2.9(2.6-3.0)	2.7(2.3–2.9)	2.9(2.8-3.0)
	Normal weight (18.5- <25 kg/m^2^)	2.8(2.6–2.8)	2.5(2.3–2.8)	2.3(2.0–3.0)	3.0(2.7-3.0)	2.8(2.4–2.9)	3.0(2.8-3.0)
	Overweight (25- <30 kg/m^2^)	2.8(2.5–2.8)	2.5(2.3–2.8)	2.3(2.0-2.7)	2.9(2.7-3.0)	2.7(2.4–2.9)	3.0(2.8-3.0)
	Obese (> 30 kg/m^2^)	2.8(2.6–2.8)	2.5(2.3–2.8)	2.5(2.0–3.0)	3.0(2.7-3.0)	2.8(2.4–2.9)	3.0(2.8-3.0)
	P value, effect size	0.018^c^*, ε^2^=0.002	0.087^c^, ε^2^=0.001	0.343^c^, ε^2^=0.000	0.681^c^, ε^2^=0.000	0.049^c^*, ε^2^=0.001	0.018^c^*, ε^2^=0.002
Residency	Rural	2.8(2.6–2.8)	2.5(2.3–2.8)	2.3(2.0–3.0)	3.0(2.7-3.0)	2.8(2.4–2.9)	3(2.8-3)
	Urban	2.7(2.5–2.8)	2.5(2.3–2.8)	2.3(2.0–3.0)	2.9(2.7-3.0)	2.7(2.3–2.9)	3(2.8-3)
	P value, effect size	0.039^b^*, r = 0.050	0.304^b^, r = 0.024	0.634^b^, r = 0.011	0.079^b^, r = 0.039	0.009^b^*, r = 0.063	0.005^b^*, r = 0.063
Faculty	Medical	2.8(2.7–2.9)	2.5(2.3-3.0)	2.3(2.3-3.0)	3.0(2.7-3.0)	2.8(2.6-3.0)	3.0(2.8-3.0)
	Non-medical	2.7(2.4–2.8)	2.3(2.0-2.8)	2.3(2.0–3.0)	2.9(2.6-3)	2.6(2.2–2.8)	2.9(2.7-3.0)
	P value, effect size	< 0.001^b^*, r = 0.383	< 0.001^b^*, r = 0.325	< 0.001^b^*, r = 0.117	< 0.001^b^*, r = 0.179	< 0.001^b^*, r = 0.339	< 0.001^b^*, r = 0.201
Academic year	1st year	2.7(2.5–2.8)	2.5(2.0-2.8)	2.3(2.0-2.7)	2.9(2.6-3.0)	2.7(2.2–2.9)	2.9(2.7-3.0)
	2nd year	2.7(2.5–2.8)	2.5(2.0-2.8)	2.3(2.0-2.7)	2.9(2.6-3.0)	2.7(2.2–2.9)	2.9(2.7-3.0)
	3rd year	2.8(2.6–2.9)	2.5(2.3–2.8)	2.7(2.3-3.0)	3(2.7-3.0)	2.8(2.4–2.9)	3.0(2.8-3.0)
	4th year	2.8(2.6–2.9)	2.5(2.3–2.8)	2.3(2.0–3.0)	3(2.7-3.0)	2.8(2.4–2.9)	3.0(2.8-3.0)
	5th year	2.8(2.6–2.9)	2.5(2.3-3.0)	2.3(2.0–3.0)	3(2.7-3.0)	2.8(2.4–2.9)	3.0(2.8-3.0)
	6th year	2.8(2.6–2.9)	2.8(2.3-3.0)	2.7(2.0–3.0)	3(2.9-3.0)	2.8(2.6-3.0)	3.0(2.9-3.0)
	P value, effect size	< 0.001^c^*, ε^2^=0.039	< 0.001^c^*, ε^2^=0.031	< 0.001^c^*, ε^2^=0.011	< 0.001^c^*, ε^2^=0.017	< 0.001^c^*, ε^2^=0.024	< 0.001^c^*, ε^2^=0.016
Father’s occupation	Government worker	2.8(2.6–2.9)	2.5(2.3–2.8)	2.3(2.0–3.0)	3.0(2.7-3.0)	2.8(2.4–2.9)	3.0(2.8-3.0)
	Private worker	2.8(2.6–2.8)	2.5(2.3–2.8)	2.3(2.0–3.0)	2.9(2.7-3.0)	2.8(2.4–2.9)	3.0(2.8-3.0)
	Unemployed	2.7(2.5–2.8)	2.5(2.0-2.8)	2.3(2.0–3.0)	2.9(2.6-3.0)	2.7(2.2–2.9)	2.9(2.7-3.0)
	P value, effect size	< 0.001^c^*, ε^2^=0.017	< 0.001^c^*, ε^2^=0.011	0.046^c^*, ε^2^=0.001	0.006^c^*, ε^2^=0.002	< 0.001^c^*, ε^2^=0.019	< 0.001^c^*, ε^2^=0.007
Father’s education	Illiterate	2.6(2.3–2.8)	2.3(2.0-2.5)	2.3(2.0-2.7)	2.9(2.4-3.0)	2.4(2.2–2.8)	2.9(2.6-3.0)
	Primary education	2.7(2.5–2.8)	2.5(2.0-2.8)	2.3(2.0–3.0)	3(2.7-3.0)	2.7(2.2–2.9)	3.0(2.8-3.0)
	Secondary education	2.8(2.5–2.8)	2.5(2.3–2.8)	2.3(2.0–3.0)	3(2.7-3.0)	2.7(2.3–2.9)	3.0(2.8-3.0)
	University education	2.8(2.6–2.8)	2.5(2.3–2.8)	2.3(2.0–3.0)	2.9(2.7-3.0)	2.7(2.4–2.9)	3.0(2.8-3.0)
	Postgraduate education	2.8(2.6–2.9)	2.5(2.3–2.8)	2.7(2.3-3.0)	3.0(2.7-3)	2.8(2.6–2.9)	3.0(2.8-3.0)
	P value, effect size	< 0.001^c^*, ε^2^=0.012	< 0.001^c^*, ε^2^=0.007	0.030^c^*, ε^2^=0.002	0.058^c^, ε^2^=0.001	< 0.001^c^*, ε^2^=0.010	0.003^c^*, ε^2^=0.004
Mother’s occupation	Government worker	2.8(2.6–2.9)	2.5(2.3–2.8)	2.3(2.0–3.0)	3.0(2.7-3.0)	2.8(2.5–2.9)	3.0(2.8-3.0)
	Private worker	2.7(2.5–2.8)	2.5(2.3–2.8)	2.3(2.0–3.0)	2.9(2.7-3.0)	2.7(2.3–2.9)	2.9(2.8-3.0)
	Housewife	2.7(2.5–2.8)	2.5(2.0-2.8)	2.3(2.0-2.7)	2.9(2.7-3.0)	2.7(2.3–2.9)	3.0(2.8-3.0)
	P value, effect size	< 0.001^c^*, ε^2^=0.013	< 0.001^c^*, ε^2^=0.005	0.011^c^*, ε^2^=0.002	< 0.001^c^*, ε^2^=0.005	< 0.001^c^*, ε^2^=0.011	< 0.001^c^*, ε^2^=0.010
Mother’s education	Illiterate	2.7(2.4–2.8)	2.3(2.0-2.8)	2.3(2.0–3.0)	2.9(2.6-3)	2.6(2.2–2.9)	2.9(2.6-3.0)
	Primary education	2.7(2.5–2.8)	2.5(2.0-2.8)	2.5(2.0–3.0)	3(2.7-3.0)	2.7(2.3–2.9)	2.9(2.8-3.0)
	Secondary education	2.8(2.5–2.8)	2.5(2.3–2.8)	2.3(2.0–3.0)	2.9(2.7-3.0)	2.7(2.4–2.9)	2.9(2.8-3.0)
	University education	2.8(2.6–2.9)	2.5(2.3–2.8)	2.3(2.0–3.0)	3(2.7-3.0)	2.8(2.4–2.9)	3.0(2.8-3.0)
	Postgraduate education	2.8(2.5–2.9)	2.5(2.3–2.8)	2.3(2.0–3.0)	3(2.7-3.0)	2.8(2.3–2.9)	3.0(2.8-3.0)
	P value, effect size	< 0.001^c^*, ε^2^=0.015	< 0.001^c^*, ε^2^=0.009	0.824^c^, ε^2^=0.001	0.012^c^*, ε^2^=0.003	< 0.001^c^*, ε^2^=0.013	< 0.001^c^*, ε^2^=0.013
Income	Enough with savings	2.8(2.6–2.9)	2.5(2.3–2.8)	2.3(2.0–3.0)	3.0(2.7-3.0)	2.8(2.4–2.9)	3.0(2.8-3.0)
	Enough without savings	2.8(2.5–2.8)	2.5(2.3–2.8)	2.3(2.0–3.0)	2.9(2.7-3.0)	2.7(2.3–2.9)	3.0(2.8-3.0)
	Not enough	2.7(2.4–2.8)	2.5(2.0-2.8)	2.3(2.0–3.0)	2.9(2.6-3.0)	2.6(2.1–2.8)	2.9(2.7-3.0)
	P value, effect size	< 0.001^c^*, ε^2^=0.012	< 0.001^c^*, ε^2^=0.005	0.345^c^, ε^2^=0.000	0.003^c^*, ε^2^=0.003	< 0.001^c^*, ε^2^=0.015	0.001^c^*, ε^2^=0.004

*Significant, ^b^: Mann-Whitney test, ^c^: Kruskal-Wallis test, r: Rank-biserial correlation, V: Cramér’s V effect size, ε^2^: Epsilon-squared, HBMS: Health Belief Model Scale.

Table 5 provides a comparison of AUSDRISK scores across various sociodemographic variables. Males had significantly higher median scores (7.0) than females (5.0) (*p* < 0.001; *r* = 0.408). The AUSDRISK score increased with age and academic year (*p* < 0.001; ε² = 0.004). There was a strong relationship between BMI and diabetes risk: underweight students had the lowest risk scores, while obese students had the highest (*p* < 0.001; ε² = 0.101). Students from urban areas had higher median risk scores than their counterparts. Enrollment in non-medical faculties, lower parental education, and unemployment were associated with increased risk. Students from households reporting insufficient income were also more likely to have higher AUSDRISK scores (median = 7.0) compared to those from more financially stable backgrounds (median = 5.0; *p* < 0.001; ε² = 0.006).

**Table 5:** Comparison of the AUSDRISK score across sociodemographic subgroups among the studied university students (N = 2787).

Studied variables		AUSDRISK score (N = 2786)			
		Median (IQR)	Mild risk, *n (%)*	Moderate risk, *n (%)*	Severe risk, *n (%)*
Gender	Female	5.0(3.0–7.0)	657(47.1%)	602(43.2%)	136(9.7%)
	Male	7.0(5.0–10.0)	285(20.5%)	765(54.9%)	343(24.6%)
	P value, effect size	< 0.001^b^*, r = 0.408	< 0.001^a^*, V = 0.303		
BMI (kg/m2)	Underweight (< 18.5 kg/m^2^)	5.0(3.0–7.0)	155(46.5%)	149(44.7%)	29(8.8%)
	Normal weight (18.5- <25 kg/m^2^)	6.0(3.0–8.0)	612(40.9%)	724(48.4%)	160(10.7%)
	Overweight (25- <30 kg/m^2^)	7.0(5.0–10.0)	141(21.6%)	351(53.9%)	159(24.4%)
	Obese (> 30 kg/m^2^)	10.0(7.0–13.0)	33(10.9%)	142(46.5%)	130(42.6%)
	P value, effect size	< 0.001^c^*,ε^2^=0.101	< 0.001^a^*, V = 0.234		
Residency	Rural	6.0(3.0–9.0)	286(38.4%)	339(45.6%)	119(16.0%)
	Urban	6.0(4.0–10.0)	656(32.1%)	1028(50.3%)	359(17.6%)
	P value, effect size	< 0.001^b^*, r = 0.077	0.008^a^*, V = 0.059		
Faculty	Medical	6.0(4.0–9.0)	576(34.6%)	848(50.9%)	241(14.5%)
	Non-medical	6.0(4.0–10.0)	366(32.6%)	519(46.2%)	237(21.2%)
	P value, effect size	0.001^b^*, r = 0.064	< 0.001^a^*, V = 0.087		
Academic year	1st year	6.0(4.0–9.0)	196(37.2%)	246(46.6%)	86(16.3%)
	2nd year	6.0(4.0–9.0)	157(31.6%)	267(54%)	71(14.4%)
	3rd year	6.0(3.0–9.0)	226(35.8%)	302(47.8%)	104(16.4%)
	4th year	6.0(4.0–9.0)	131(33.1%)	198(50.2%)	66(16.7%)
	5th year	7.0(4.0–10.0)	181(32%)	262(46.5%)	121(21.5%)
	6th year	6.0(4.0–9.0)	52(30.0%)	91(52.3%)	31(17.7%)
	P value, effect size	0.013^c^*, ε^2^=0.004	0.059^a^, V = 0.056		
Father’s occupation	Government worker	6.0(4.0–9.0)	451(35.5%)	615(48.4%)	204(16.1%)
	Private worker	6.0(4.0–9.0)	265(33%)	396(49.2%)	144(17.8%)
	Unemployed	6.0(4.0–10.0)	225(31.7%)	355(50.0%)	130(18.3%)
	P value, effect size	0.012^c^*, ε^2^=0.002	0.387^a^, V = 0.027		
Father’s education	Illiterate	8.0(6.0–11.0)	21(18.2%)	62(52.8%)	34(29.0%)
	Primary education	7.0(4.0–10.0)	81(34.1%)	112(47.2%)	45(18.7%)
	Secondary education	6.0(4.0–9.0)	227(35.7%)	327(51.3%)	83(13.1%)
	University education	6.0(3.0–9.0)	392(34.0%)	557(48.4%)	202(17.6%)
	Postgraduate education	6.0(4.0–9.0)	220(34.2%)	308(48.0%)	114(17.8%)
	P value, effect size	< 0.001^c^*, ε^2^=0.006	0.001^a^*, V = 0.070		
Mother’s occupation	Government worker	6.0(3.0–9.0)	309(37.3%)	386(46.5%)	134(16.2%)
	Private worker	7.0(4.0–12.0)	583(32.6%)	913(51.0%)	293(16.4%)
	Housewife	6.0(4.0–9.0)	49(29.5%)	67(40.2%)	51(30.2%)
	P value, effect size	0.013^c^*, ε^2^=0.000	< 0.001^a^*, V = 0.071		
Mother’s education	Illiterate	7.0(5.0–10.0)	65(20.9%)	172(55%)	75(24.1%)
	Primary education	6.0(4.0–10.0)	117(31.0%)	186(49.5%)	74(19.6%)
	Secondary education	6.0(3.0–8.0)	295(39.1%)	360(47.9%)	98(13.0%)
	University education	6.0(3.0–9.0)	360(35.7%)	495(49.0%)	154(15.3%)
	Postgraduate education	6.0(4.0–10.0)	105(31.3%)	154(45.7%)	77(22.9%)
	P value, effect size	< 0.001^c^*, ε^2^=0.020	< 0.001^a^*, V = 0.098		
Income	Enough with savings	6.0(4.0–9.0)	367(34.7%)	506(47.9%)	183(17.3%)
	Enough without savings	6.0(4.0–9.0)	474(34.8%)	678(49.8%)	209(15.3%)
	Not enough	7.0(4.0–10.0)	101(27.4%)	182(49.3%)	86(23.4%)
	P value, effect size	< 0.001^c^*, ε^2^=0.006	0.003^a^*, V = 0.054		

*Significant, ^a^: Chi-squared test, ^b^: Mann-Whitney test, ^c^: Kruskal-Wallis test, r: Rank-biserial correlation, V: Cramér’s V effect size, ε^2^: Epsilon-squared, AUSDRISK: Australian Type 2 Diabetes Risk Assessment Tool.

N.B. AUSDRISK scores were weighted by sex to ensure balanced representation when comparing across sociodemographic subgroups.

**Table 6:** Multiple linear regression analyses predicting diabetes knowledge (DKQ-18), health belief model scores (HBMS), and AUSDRISK scores based on sociodemographic and personal characteristics of the study participants.

Studied variables		DKQ-18 score	HBMS score	AUSDRISK score
		β (P-value)	β (P-value)	β (P-value)
Age (Years)		0.043(0.146)	0.017(0.591)	0.123(< 0.001*)
Gender (Ref: Female)	Male	-0.066 (< 0.001*)	-0.106(< 0.001*)	0.305(< 0.001*)
BMI (kg/m2)		0.010 (0.567)	-0.004(0.819)	0.284(< 0.001*)
Country (Ref: Egypt)	Saudi Arabia	-0.140 (< 0.001*)	-0.106(< 0.001*)	0.035(0.117)
	Yemen	-0.047 (0.038*)	-0.099(< 0.001*)	0.080(0.001*)
Residency (Ref: Urban)	Rural	0.009 (0.640)	0.001(0.969)	-0.015(0.437)
Faculty (Ref: Medical)	Non-medical	-0.411 (< 0.001*)	-0.228(< 0.001*)	0.066(< 0.001*)
Academic year		0.155 (< 0.001*)	0.152(< 0.001*)	-0.061(0.031*)
Father’s occupation (Ref: Government worker)	Private worker	0.027 (0.155)	0.017(0.409)	-0.004(0.820)
	Unemployed	-0.024 (0.211)	-0.056(0.007*)	0.008(0.666)
Father’s education (Ref: University education)	Illiterate	-0.068 (< 0.001*)	-0.041(0.040*)	0.035(0.066)
	Primary education	-0.053 (0.005*)	0.004(0.828)	-0.016(0.408)
	Secondary education	-0.004 (0.838)	0.009(0.667)	-0.009(0.634)
	Postgraduate education	0.047 (0.021*)	0.052(0.018*)	-0.027(0.188)
Mother’s occupation (Ref: Housewife)	Government worker	0.052 (0.012*)	0.028(0.207)	0.025(0.225)
	Private worker	0.004 (0.801)	-0.019(0.315)	0.056(0.001*)
Mother’s education (Ref: University education)	Illiterate	0.039 (0.084)	-0.030(0.211)	0.031(0.191)
	Primary education	-0.003 (0.881)	0.004(0.874)	0.001(0.945)
	Secondary education	0.004 (0.841)	-0.016(0.483)	-0.039(0.071)
	Postgraduate education	-0.048 (0.017*)	-0.073(0.001*)	0.040(0.048*)
Income (Ref: Enough without savings)	Enough with savings	0.042 (0.019*)	0.014(0.446)	0.000(0.985)
	Not enough	-0.001 (0.940)	-0.044(0.022*)	0.027(0.137)

*Significant β: Standardized coefficient, DKQ-18: Diabetes Knowledge Questionnaire-18, HBMS: Health Belief Model Scale, AUSDRISK: Australian Type 2 Diabetes Risk Assessment Tool.

Table 6 presents the results of the multiple linear regression analyses predicting diabetes knowledge, health beliefs, and diabetes risk using the AUSDRISK tool. For diabetes knowledge (DKQ-18), male gender (β = -0.066), and being in a non-medical faculty (β = -0.411) were independently associated with lower scores (*p* < 0.001). Higher academic year (β = 0.155), higher father education (β = 0.047), and household income (β = 0.042) were positively significant predictors. For health beliefs (HBMS), male gender (β = -0.106), non-medical faculty (β = -0.228), were associated with lower HBMS scores, while higher academic year predicted higher scores (β = 0.152). Regarding diabetes risk (AUSDRISK), male gender (β = 0.305), older age (β = 0.123), higher BMI (β = 0.284), and Yemeni nationality (β = 0.080) were positively associated with risk. On the other hand, the academic year had a negative association with risk (β = -0.061). Faculty type and parental education were also significant predictors in the models.

## Discussion

University students represent a critical yet often overlooked population at risk of developing metabolic disorders, including T2DM. This vulnerability stems from a convergence of lifestyle-related risk factors and psychosocial stressors. During the transition to adulthood, students frequently adopt unhealthy behaviours such as poor dietary habits, sedentary routines, irregular sleep patterns, and persistent academic stress, which contribute to increased diabetes risk^32^. These behaviours are often compounded by both modifiable risk factors, such as physical inactivity and smoking, and non-modifiable ones like family history of diabetes^33^. The findings of the present study revealed significant inter-country differences in diabetes-related knowledge, beliefs, and risk profiles. Egyptian students demonstrated significantly higher diabetes knowledge and stronger health beliefs than their Saudi and Yemeni counterparts, whereas Yemeni students showed the highest T2DM risk based on AUSDRISK scores. These inter-country variations parallel the well-documented regional burden of T2DM, as Arab coountries continues to exhibit one of the highest global diabetes prevalences. The elevated risk observed among students particularly in Yemen reflects the early emergence of metabolic risk factors that contribute to the region’s accelerating diabetes epidemic.

### Diabetes knowledge

This study revealed notable differences in diabetes knowledge among university students across Egypt, Saudi Arabia, and Yemen. Egyptian students demonstrated the highest proportion of good knowledge, followed by those from Saudi Arabia and Yemen. These inter-country variations likely reflect disparities in public health education systems, access to information, and national investment in awareness campaigns.

Sociodemographic factors were significantly associated with knowledge levels. Students in higher academic years, those enrolled in medical faculties, rural residents, and individuals from households with higher income and parental education showed significantly better diabetes knowledge. These findings are supported by Ali et al. (2023), who found that 70.6% of women in Sohag, Egypt, had satisfactory diabetes knowledge, particularly those with higher education, older age, and a family history of diabetes^34^. Similarly, Al-Aboudi et al. (2021) reported that 60% of nursing students in Saudi Arabia had moderate knowledge levels, with education, income, and employment being influential factors^35^. The lower knowledge levels observed among Yemeni students may reflect broader socioeconomic and infrastructural challenges, including limited health education initiatives and restricted access to preventive services. Prior research has shown that countries investing more in public health education tend to achieve higher public awareness^36,37^. Financial stability also emerged as a predictor of knowledge, supporting the notion that individuals from higher-income households are more likely to access health-related information and resources^38^. Gender differences were evident, with female students exhibiting significantly higher diabetes knowledge. This aligns with previous findings by Al-Aboudi et al. (2021), who reported greater perceived and actual knowledge among female nursing students^35^. Additionally, medical faculty enrollment was a strong predictor of better knowledge, echoing the results of Fekry et al. (2021), who found that senior medical students in Egypt demonstrated higher levels of health knowledge and academic competence^39^.

### Beliefs and attitudes toward diabetes prevention and management

The present study revealed notable differences in diabetes-related health beliefs among students across the three countries, with significant variation observed in perceived barriers, susceptibility, and engagement in health-promoting behaviors. Students from Saudi Arabia and Egypt consistently reported higher perceived benefits and fewer perceived barriers compared to their Yemeni peers. This may reflect the influence of more structured public health campaigns, stronger healthcare systems, and better access to diabetes education in these countries^36,37^.

Interestingly, perceptions of the seriousness of diabetes were similar across all three populations, suggesting a shared recognition of the condition’s severity. This uniformity is consistent with broader global trends that highlight growing public awareness of diabetes as a serious, life-altering disease^40^. The widespread availability of health information, through social media, digital platforms, and international awareness campaigns, likely plays a key role in reinforcing this understanding.

Health beliefs also differed by individual characteristics. Female students demonstrated significantly stronger beliefs related to diabetes prevention and management, aligning with existing evidence that women often show higher levels of health awareness and risk perception^35^. Students enrolled in medical faculties and those in advanced academic years also reported higher scores across most belief dimensions, which may reflect their greater exposure to health-related information and deeper understanding of disease prevention strategies^41^. Additionally, students from households with higher income or more educated parents tended to report stronger health beliefs, findings that highlight the influence of socioeconomic factors on health attitudes and behaviors^38^.

### Diabetes risk among undergraduates

To evaluate the risk of developing T2DM, various risk assessment tools have been developed for identifying both undiagnosed (prevalent) and incident cases. Among these, the Finnish Diabetes Risk Score (FINDRISC), the Peruvian Risk Score, and AUSDRISK have demonstrated efficacy across diverse populations^42–44^. In this study, the AUSDRISK tool was employed to assess diabetes risk among university students in Egypt, Saudi Arabia, and Yemen.

Our results revealed significant inter-country differences. Yemeni students exhibited the highest median AUSDRISK scores, with 22.0% categorized as high-risk. This elevated risk may be attributed to compounding sociopolitical and economic challenges, including conflict, nutritional insecurity, and limited access to preventive healthcare factors known to heighten chronic disease burden in low-resource settings^45^. In contrast, Saudi Arabia had the highest proportion of students classified at mild risk (43.2%), likely reflecting stronger public health infrastructure, routine screening, and active awareness campaigns. Egyptian participants showed the highest proportion in the moderate-risk category (53.9%), indicating a pressing need for early intervention programs to halt progression to higher risk levels. Comparable findings were reported by Farag et al. (2023), who noted that 39.4% and 39.1% of participants scored in the moderate and high-risk categories, respectively, using the Arabic version of AUSDRISK^26^, while only 21.5% were at mild risk. Other studies within Egypt and internationally have similarly found high rates of T2DM risk, with age identified as a key determinant^46–49^. Younger populations tend to show lower risk due to the age-dependent nature of the AUSDRISK score. This was evident in studies among Australian university students and among Malaysian students, as well as Egyptian students enrolled at Tanta University, where AUSDRISK proved both feasible and effective for youth-based screening^50,51^. Findings are further supported by regional data. In Yemen, the FINDRISC tool applied to 176 senior medical students revealed that 22.7% had slightly elevated risk, while only 2.3% and 0.6% were classified as moderate and high risk, respectively^52^. Similar patterns were observed in Nepal and Jordan, where 22.2% and 26.2% of medical students had slightly elevated risk levels^53,54^. In Saudi Arabia, Abd El-Razik et al. (2023) reported that 61.9% of 740 university students were at moderate to high risk^55^, consistent with a systematic review study, which emphasizes the growing burden of metabolic risk factors among youth^56^. A recent study by Alshaikh et al. (2024) found that 46.2% of Saudi university students scored above 9 on the AUSDRISK, indicating likely progression to impaired glucose tolerance within five years^57^. Notably, 21.9% scored ≥ 13, suggesting a high probability of developing T2DM. These findings align with projections by Bernabe-Ortiz et al. (2016), who forecast a sharp rise in diabetes prevalence in Saudi Arabia—from 32.8% in 2015 to 45.36% by 2030^58^. These results underscore an alarming trend in T2DM risk among university students in the region. The rising burden among young adults highlights the urgent need for early screening initiatives and culturally tailored lifestyle interventions to mitigate long-term complications and reduce healthcare burdens.

### Strengths and limitations

This study is one of the first to comprehensively assess diabetes-related knowledge, health beliefs, and risk using validated tools (DKQ-18, HBMS, and AUSDRISK) among a large, diverse sample of university students across three Arab countries with differing income levels. The multicenter design and inclusion of both medical and non-medical faculties enhance the generalizability of findings. Despite these strengths, the study has several limitations. Its cross-sectional design limits causal inference between sociodemographic factors and diabetes knowledge, beliefs, or risk. The use of self-reported data may introduce reporting bias. Moreover, the AUSDRISK tool provides a risk assessment rather than a confirmed prediction tool for T2DM diabetes. Additionally, the purposive sampling method, while stratified, may limit the representativeness of the broader student populations in each country.

## Conclusion

This multinational study highlights significant disparities in diabetes-related knowledge, health beliefs, and risk among university students across Egypt, Saudi Arabia, and Yemen. Students in Egypt demonstrated the highest levels of diabetes knowledge and health-promoting beliefs, whereas Yemeni students exhibited the highest diabetes risk. Medical education, advanced academic standing, female gender, and favorable socioeconomic factors were consistently associated with better knowledge and beliefs. In contrast, male gender, higher BMI, older age, non-medical faculty enrollment, and socioeconomic disadvantage were associated with elevated diabetes risk. The findings support tailored recommendations at both national and regional levels, with priorities such as strengthening basic health education and improving access to preventive services, particularly in lower-income countries. Regionally, coordinated youth-focused strategies are needed that account for differences in health system capacity and socio-economic conditions across Arab countries. These context-specific, preventive strategies within university settings should particularly target male students, those in non-medical disciplines, and individuals from lower-income backgrounds. Integrating diabetes education into academic curricula, promoting campus-wide health campaigns, and addressing structural barriers to preventive care may collectively enhance awareness, shift health beliefs, and reduce future disease burden in this vulnerable population.

## Supplementary Information

Below is the link to the electronic supplementary material.

Supplementary Material 1

## Data Availability

All data are available upon request from the first author.

## Abbreviations

AUSDRISK: Australian type 2 diabetes risk assessment tool
BMI: Body mass index
DKQ-18: Diabetes knowledge questionnaire-18
DM: Diabetes mellitus
FINDRISC: Finnish diabetes risk score
HBMS: Health belief model scale
IDF: International Diabetes Federation
NCD: Non-communicable disease
T2DM: Type 2 diabetes mellitus
WHO: World Health Organization
