# Supplementary material for: Risk assessment for type 2 diabetes mellitus and its association with knowledge and health beliefs among university students in three Arab countries
Source: Sci Rep. 2026 Mar 27;16:10367. doi: 10.1038/s41598-026-41511-5 (PMC13031495; doi:10.1038/s41598-026-41511-5)
Supplement: Supplementary file 1 — Supplementary Material 1 [file 41598_2026_41511_MOESM1_ESM.docx]

**Table S1: Items of the Validated Arabic Version of AUSDRISK**

| Question | | Points |
| --- | --- | --- |
| 1. Age | | |
| Less than 35 years | | 0 |
| 35–44 years | | 2 |
| 45–54 years | | 4 |
| 55–64 years | | 6 |
| 65 years or older | | 8 |
| 2. Gender | | |
| Female | | 0 |
| Male | | 3 |
| 3. Has either of your parents or siblings been diagnosed with diabetes (type 1 or type 2)? | | |
| No | | 0 |
| Yes | | 3 |
| 4. Have you ever had high blood sugar levels (e.g., during a health checkup, illness, or pregnancy)? | | |
| No | | 0 |
| Yes | | 6 |
| 5. Currently, are you taking medication for high blood pressure? | | |
| No | | 0 |
| Yes | | 2 |
| 6. Currently, do you smoke cigarettes or any other tobacco product daily? | | |
| No | | 0 |
| Yes | | 2 |
| 7. Typically, how often do you consume vegetables or fruits? | | |
| Every day | | 0 |
| Not every day | | 1 |
| 8. On average, do you believe you engage in physical activity for at least 2.5 hours per week (e.g., 30 min daily for 5 days or more per week)? | | |
| Yes | | 0 |
| No | | 2 |
| 9. Waist measurement at the bottom of the ribs (usually at the level of the navel and while standing): | | |
| Waist measurement (cm) | | |
| Men | Women |  |
| Less than 102 cm | Less than 88 cm | 0 |
| 102–110 cm | 88–100 cm | 4 |
| More than 110 cm | More than 100 cm | 7 |

AUSDRISK: Australian Type 2 Diabetes Risk Assessment Tool.

**Table S2. Country-Based Differences in AUSDRISK Components and Scores among the Studied University Students (N=2787)**

| **AUSDRISK tool** | | | **Total** | **Egypt** | **Saudi Arabia** | **Yemen** | **P-value** |
| --- | --- | --- | --- | --- | --- | --- | --- |
|  |  |  | **N= 2787** | **N= 1153** | **N= 963** | **N= 671** |  |
| **Items** | | **Point** |  |  |  |  |  |
| **Gender** | **Female** | **0** | 2088(74.9%) | 859(75.0%) | 888(90.1%) | 341(52.0%) | **<0.001*^a^** |
|  | **Male** | **3** | 699(25.1%) | 286(25.0%) | 98(9.9%) | 315(48.0%) |  |
| **Have you ever been found to have high blood glucose (sugar)** | **No** | **0** | 1729(62%) | 766(66.9%) | 587(59.5%) | 376(57.3%) | **<0.001*^a^** |
|  | **Yes** | **6** | 1058(38%) | 379(33.1%) | 399(40.5%) | 280(42.7%) |  |
| **Have either of your parents or any of your brothers or sisters been diagnosed with diabetes** | **No** | **0** | 2460(88.3%) | 1040(90.8%) | 839(85.1%) | 581(88.6%) | **<0.001*^a^** |
|  | **Yes** | **3** | 327(11.7%) | 105(9.2%) | 147(14.9%) | 75(11.4%) |  |
| **Are you currently taking medication for high blood pressure?** | **No** | **0** | 2688(96.4%) | 1115(97.4%) | 952(96.6%) | 621(94.7%) | **0.011*^a^** |
|  | **Yes** | **3** | 99(3.6%) | 30(2.6%) | 34(3.4%) | 35(5.3%) |  |
| **Do you currently smoke cigarettes or any other tobacco products daily?** | **No** | **0** | 2627(94.3%) | 1102(96.2%) | 943(95.6%) | 582(88.7%) | **<0.001*^a^** |
|  | **Yes** | **2** | 160(5.7%) | 43(3.8%) | 43(4.4%) | 74(11.3%) |  |
| **How often do you eat vegetables or fruits?** | **Every day** | **0** | 1278(45.9%) | 620(54.1%) | 387(39.2%) | 271(41.3%) | **<0.001*^a^** |
|  | **Not every day** | **2** | 1509(54.1%) | 525(45.9%) | 599(60.8%) | 385(58.7%) |  |
| **On average, would you say you do at least 2.5 hours of physical activity per week?** | **Yes** | **0** | 1425(51.1%) | 624(54.5%) | 461(46.8%) | 340(51.8%) | **0.002*^a^** |
|  | **No** | **2** | 1362(48.9%) | 521(45.5%) | 525(53.2%) | 316(48.2%) |  |
| **Waist circumference** | **Male < 102 cm/female < 88 cm** | **0** | 1635(58.7%) | 665(58.1%) | 602(61.1%) | 368(56.1%) | **0.351^a^** |
|  | **Male 102–110 cm/female 88–100 cm** | **4** | 1017(36.5%) | 423(36.9%) | 341(34.6%) | 253(38.6%) |  |
|  | **Male > 110 cm/female > 100** | **7** | 135(4.8%) | 57(5.0%) | 43(4.4%) | 35(5.3%) |  |

*Significant, a: chi-squared test, AUSDRISK: Australian Type 2 Diabetes Risk Assessment Tool.

N.B. values are presented as Number (%).
